# Effect of resistance exercise on stress, lower extremity edema, and body composition in intensive care unit nurses

**DOI:** 10.1097/MD.0000000000032358

**Published:** 2022-12-30

**Authors:** Ki Yong Kim, Won Jong Kim

**Affiliations:** a College of health care, Gimcheon University, Gyeongsangbuk-do, Republic of Korea; b College of Nursing, Eulji University, Uijeongbu-si, Gyeonggi-do, Republic of Korea.

**Keywords:** body composition, edema, exercise, intensive care unit, stress

## Abstract

This study investigated the effects of resistance exercises using elastic bands on stress, lower extremity edema, and body composition in intensive care unit nurses.

**Methods:**

Twenty-three and 21 participants were classified into the experimental and control groups, respectively. Only the experimental group was subjected to a resistance exercise program using elastic bands for 8 weeks. Variables were measured before the experimental treatment and at the 4th and 8th weeks post-treatment. Stress was assessed using a numeric rating scale and stress index. Lower extremity edema was determined using a tape measure. Body composition around the calf and tibia muscle was measured using a body composition analyzer. The homogeneity of participants’ general characteristics and the dependent variable was ensured.

**Results:**

Following experimental treatment, subjective (*F* = 11.674, *P* < .001) and objective stresses (*F* = 6.965, *P* < .001) decreased. No difference was detected in calf and ankle circumference between the groups, while differences in muscle thickness (left, *F* = 31.708, *P* < .001; right, *F* = 18.630, *P* < .001) and fat thickness (left, *F* = 19.984, *P* < .001; right, *F* = 24.640, *P* < .001) were observed. Muscle thickness increased, and fat thickness decreased in the body composition around the lower extremities.

**Conclusion:**

Resistance exercises using the TheraBand can be an intervention to decrease stress and improve lower extremity body composition in intensive care unit nurses.

## 1. Introduction

### 1.1. Study rationale

Work characteristics that cause poor body mechanics, and prolonged standing, place nurses at an increased risk of developing musculoskeletal disorders. Most nurses experience constant physical and mental stress from excessive job demands during work hours.^[1]^ While on duty, nurses frequently perform heavy physical tasks requiring unstable postures. These include positioning patients or computerized tasks such as electronic medical record updates and entering orders into the order communication system.^[2]^ Intensive care unit (ICU) patients are incapable of changing their body positions and therefore completely reliant on nurses, requiring comprehensive nursing intervention. Prolonged standing and increased consumer healthcare demands, associated with addressing patient’s diverse nursing requirements, place ICU nurses under more significant psychological and physical stress compared to nurses in other settings.^[3,4]^

While an appropriate stress level may positively impact nursing work, excessive stress is detrimental to physical and mental health, leading to a deterioration in the quality of care and performance.^[5,6]^ Continuous stress stimulates the hypothalamus and induces hyper-reactivity of the autonomic nervous system, increasing cortisol secretion, resulting in an increased heart rate, raised blood pressure, and mood changes such as depression, anxiety, and anger.^[7]^

Prolonged standing during shifts, which stagnates blood flow and hinders circulation in the lower extremities, leading to persistent health problems such as lower extremity edema, causes substantial physical stress in clinical nurses.^[8]^ Lower extremity edema and pain cause vascular diseases, fatigue, and stress in nurses.^[9,10]^ Prolonged work while standing causes continuous muscle pain with an elevated risk of tissue injuries and stagnated circulation, negatively altering the body composition by decreasing muscle mass and increasing fat.^[11,12]^ Reduced muscle mass due to prolonged standing hinders appropriate body alignment, undermines mobility, deteriorates cognitive functions and quality of life, and causes depression.^[13]^ Furthermore, adverse changes in body composition, characterized by increased fat and consequent obesity, can induce cardiovascular diseases and metabolic syndrome.^[14,15]^ Many intervention studies have been conducted among workers with prolonged standing to improve stress, lower extremity edema, and imbalance in body composition. Studies have reported that elastic compression stockings,^[8]^ taping,^[9]^ aromatherapy foot massages,^[16]^ and ankle pump exercises^[17]^ positively influence stress and lower extremity edema. Of the various interventions, elastic compression stockings are beneficial in terms of their noninvasiveness and applicability regardless of the work environment, and they have been reported to reduce lower extremity edema effectively.^[18]^

Exercise increases muscle mass, reduces body fat, and decreases the risk of adverse events compared to pharmacological therapy.^[19,20]^ Resistance exercises have been reported to induce positive physiological and chemical changes in the human body. Benefits include reducing body weight and body fat, increasing muscle mass, improving fitness and blood circulation, and alleviating stress, thereby promoting physical and mental health and preventing various diseases.^[21,22]^ Resistance exercises strengthen the muscles by stimulating muscle fibers and activating nerve fibers in the muscles.^[23]^ However, inappropriate resistance exercises that do not consider an individuals’ muscular durability strain the musculoskeletal system and may elevate the risk of secondary joint injuries.^[24]^ Furthermore, resistance exercise using an elastic band refers to a type of exercise that induces isometric and isotonic contractions by loading the muscles using the band’s elasticity because muscle loading can be adjusted and tailored to an individual’s needs. Additionally, it safely and effectively increases muscle mass and strength, with a low risk of secondary injury.^[25]^ Resistance exercise using an elastic band can be performed in any place and at any time at a low cost, and it has been reported to be effective in increasing body muscle mass, reducing body fat, and relieving stress.^[26–28]^ Therefore, resistance exercises using an elastic band are deemed appropriate for reducing stress, lowering extremity edema, and improving body composition in ICU nurses.

### 1.2. Objective

To investigate the effects of an 8-week elastic band exercise regimen on stress, lower extremity edema, and body composition in ICU nurses.

### 1.3. Study hypotheses

The study hypotheses are as follows:

1) Hypothesis 1: There will be a difference in stress between the experimental group that performed the elastic band exercise and the control group.

*Subhypothesis 1.1*: There will be a difference in subjective stress between the experimental group that performed elastic band exercise and the control group.

*Subhypothesis 1.2*: There will be a difference in objective stress between the experimental group that performed the elastic band exercise and the control group.

2) Hypothesis 2: There will be a difference in lower extremity edema between the experimental group that performed elastic band exercises and the control group.

*Subhypothesis 2.1*: There will be a difference in calf circumference between the experimental group that performed elastic band exercises and the control group.

*Subhypothesis 2.2*: There will be a difference in ankle circumference between the experimental group that performed elastic band exercises and the control group.

3) Hypothesis 3: There will be a difference in body composition between the experimental group that performed elastic band exercises and the control group.

*Subhypothesis 3.1:* There will be a difference in muscle thickness between the experimental group that performed elastic band exercises and the control group.

*Subhypothesis 3.2:* There will be a difference in fat thickness between the experimental group that performed elastic band exercises and the control group.

## 2. Methods

### 2.1. Study design

**Figure 1.**
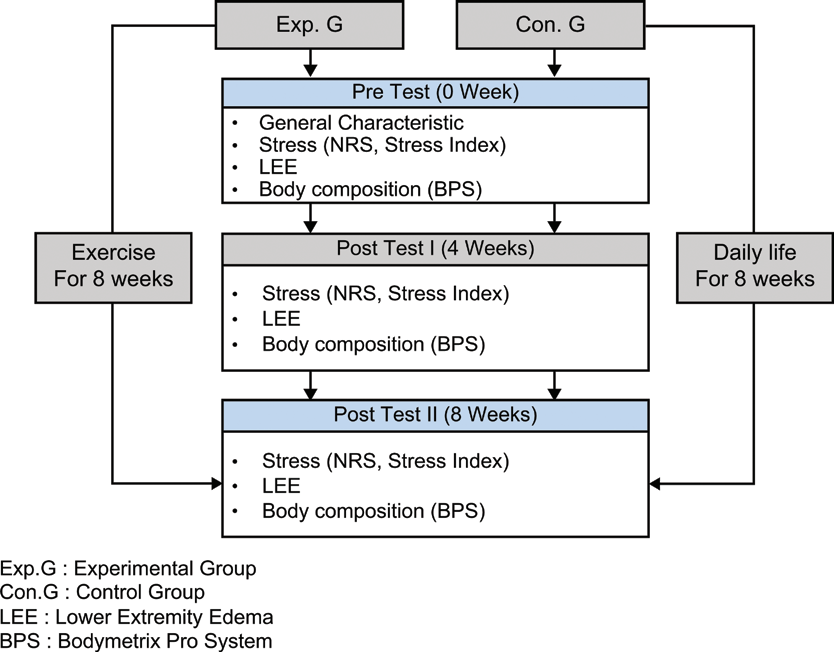
Research design. NRS = numeric rating scale. LEE = lower extremity edema. BPS = bodymetrix pro system.

The authors used an experimental study design that used a randomized pretest-posttest non-synchronized design with a control group (KCT0005890). The study aimed to investigate the effects of an 8-week elastic band exercise program on stress, lower extremity edema, and body composition in ICU nurses (Fig. 1).

### 2.2. Study participants

#### 2.2.1. Sample size determination.

**Figure 2.**
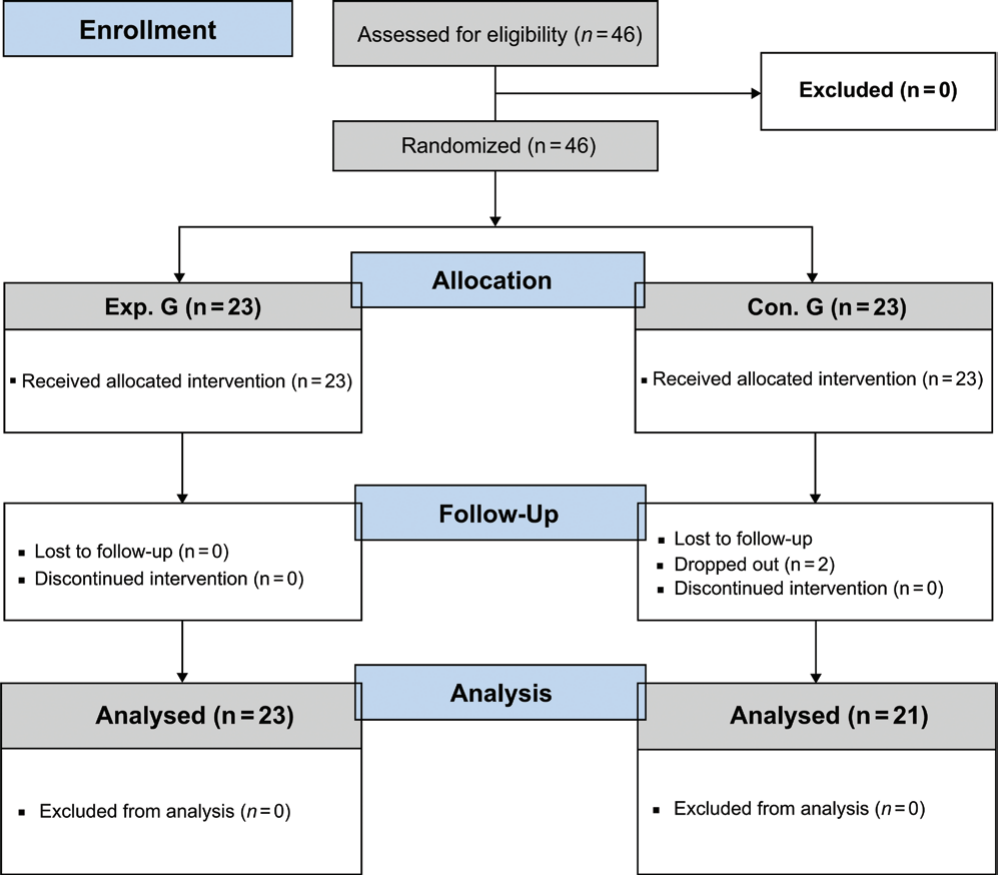
Process flow diagram.

The sample size was determined using the G-power 3.1.9.7 software. The effect size in a previous study^[29]^ for comparing between 2 groups was 0.56, showing a high effect of the exercise. Based on this, we calculated the sample size for a comparison between 2 groups to require a minimum sample size of 19 for each group, for an effect size of 0.05, power of 0.95, 2 groups, 3 measurement variables, and a correlation coefficient of 0.5. Considering a potential withdrawal rate of 20% by bearing in mind the 8-week regimen and the characteristics of the study population (turnover, interdepartmental transfer),^[30]^ 46 participants were recruited by the announcement. They were randomized to the experimental and control groups, with 23 in each group. Two participants withdrew from the study; therefore, data from 23 participants in the experimental group and 21 in the control group were included in the analysis. There were no significant differences in the general characteristics of the withdrawn and included participants (Fig. 2).

#### 2.2.2. Participant selection.

The Institutional Review Board approved this study of E University (EUN20-020) before data collection. Participants were ICU nurses working in E University Hospital in D City who provided written informed consent. The recruitment period was from June 29 to July 10, 2020. Recruitment announcement posters were posted on groupware bulletin boards and ICU bulletin boards. The inclusion criterion was nurses aged 20 to 64 years who provided informed consent to participate in the study. The exclusion criteria were history of musculoskeletal surgery in the lower limbs within the past year, an open wound or skin disease in the feet or legs, vascular or musculoskeletal disorders, anatomical anomalies, pregnancy, parturition in the past 6 months, and use of joint-related medications (e.g., steroids and analgesics).

#### 2.2.3. Participant allocation and concealment.

After the participants selected and listed numbered tables in the box, they set “0” as the experimental group and “1” as the control group in Microsoft Excel version 2016 (Microsoft Corp., Redmond, WA) and applied the random number generation method. Half of the participants were randomly assigned to the experimental group (N = 23) and half to the control group (N = 23), and data were 1^st^ collected from the control group. As for the control group, 2 out of 23 participants were eliminated, and 21 participants were used in the analysis. All 23 participants in the experimental group were used in the analysis.

### 2.3. Experimental treatment

#### 2.3.1. Experimental tools.

▫ Composition of the elastic band exercise program

An 8-week elastic band resistance training regimen (exercise program) was developed based on the elastic band exercises of the resistance training program developed by the National Sports Promotion Corporation^[31]^ and exercises performed in a previous study.^[32]^ The appropriateness of the program was evaluated by 1 orthopedic surgeon, 1 leisure sports instructor, 1 adult nursing professor, and 2 clinical nurses. The program consisted of 5 leg exercises.

Resistance exercises were chosen because they effectively facilitate the recovery of muscular endurance and improve calf function by increasing the strength and stability of the surrounding tissues. An elastic band was chosen as the exercise tool because it can be used for resistance exercises in all ages, is easy to use, has a low risk of physical injury, and can be used anywhere.^[33]^

The exercise duration was set to 48 hours in consideration of the fibrosis of the calf tissues and muscle atrophy^[34]^; hence, the program was set to 3 sessions per week. Five resistance band exercises were included: seated foot arch; heel raise; lunge; tibialis anterior exercise; and lying leg curl. The intensity of the exercise was determined based on the durability of the individuals. Durability was measured using a red band, which is standard for adult women. An individual’s durability was assessed by having the participants perform ten repetitions of seated foot arch with 2.5 kg of loading with the elastic band stretched at 100%. A yellow elastic band was used instead for the participant who felt that their muscles were strained using the red elastic band. The participants performed ten repetitions of the seated foot arch, and the color of the band (intensity of exercise) was chosen based on the number of repetitions they were able to perform: level 2 (red) for < 10 repetitions; level 3 (green) for ten repetitions; and level 4 (blue) for > 10 repetitions.

Based on a previous study,^[25]^ 1 cycle comprised 15 repetitions of each of the 5 exercises, and 5 cycles were performed. Exercises 1 and 2 were performed on the left and right sides simultaneously and exercises 3, 4, and 5 were performed for the left and right sides. Thus, 1 cycle was completed in approximately 7 minutes. The elastic band exercise program consisted of 3 sessions per week (Mondays, Wednesdays, Fridays), with 5 sets per session. Warm-up and cool-down exercises were performed before and after the program, respectively. Each session lasted 45 minutes, with a 5-minute warm-up, 5-minute cool-down stretching, and 5-minute rest. Thus, the entire program lasted 60 minutes.

#### 2.3.2. Elastic band exercise protocol.

The researchers learned how to perform the resistance exercise program developed based on previous studies^[31,32]^ from a leisure sports instructor; then, they demonstrated the exercises to the participants. They also developed a video showing the exercise protocol with the assistance of a leisure sports instructor, and a video and a booklet were provided to the participants. All the above steps were conducted to ensure that all participants in the experimental group performed the same exercise regimen. On the exercise day, the researcher directly encouraged the experimental group to perform the exercise using the social network service group chat (Kakaotalk, Kakao Corp, Jeju-do, Republic of Korea); compliance with the sessions was marked on a checklist. The control group followed their normal daily life routines without participating in an exercise intervention.

### 2.4. Study instruments

#### 2.4.1. Stress.

Subjective stress was measured using the numeric rating scale (NRS). The participants were instructed to indicate their stress level on a line having 0 on the left end and 10 on the right end, with a higher score indicating a higher level of stress.

Objective stress was measured using the stress index (SI), based on heart rate variability (HRV) measured with a pulse oximeter (Canopy 9 RSA, IEMBIO Co., Ltd., Gangwon-do, Korea) among the indicators for evaluating autonomic nervous system (ANS) activity. To compute the SI, the pulse rate was measured for 5 minutes using an oximeter, and HRV and ANS balance were analyzed and quantified using standard leads. The SI ranges from 0 to 10, with a higher index indicating greater exposure to stress.

#### 2.4.2. Lower extremity edema.

Changes in lower extremity edema were measured using a tape measure. In this study, a fiberglass measuring tape (PIE, BagelLabs Co., Ltd., Korea) was used to measure the calf and ankle circumferences to evaluate lower extremity edema. Lower extremity edema was measured in the following sites: Calf circumference: The circumference (cm) of the thickest part of the calf to the first decimal point was measured with the participant sitting on a chair with their knees raised at 90°. Ankle circumference: The circumference (cm) of the medial malleolus of the inferior part of the tibia and the lateral malleolus of the inferior part of the fibula were measured to the first decimal point with the participant sitting on a chair with the knees raised at 90°.

#### 2.4.3. Body composition.

▫ Body composition analyzer (bodymetrix pro system)

This study used a body composition analyzer that measures muscle and fat thickness via noninvasive ultrasound (Bodymetrix Pro System, Intelametrix, Livermore) to measure tissue thickness. The probe was placed on the belly of the muscle of interest, and ultrasound was emitted, producing strong signals at the boundaries of the skin, fat, skeletal muscle, and bones. The fat and muscle thickness in the corresponding sites were measured based on the peak signals. Measurements were taken at the gastrocnemius and tibialis anterior. The sum of the distance from the skin to the superficial adipose tissue and the distance from the end of the superficial adipose tissue to the deep adipose tissue was defined as the fat thickness (mm), whereas the distance from the end of the deep adipose tissue to the muscle tissue was defined as the muscle thickness (mm).

▫ Measurement of the lower extremity body composition

This study measured body composition in the gastrocnemius muscle (measurement was taken at the point of the greatest calf circumference perpendicular to the protruding medial epicondyle) and tibialis anterior (measurements were taken at the point of the greatest calf circumference perpendicular to the protruding lateral tibial condyle).

### 2.5. Data collection

Data for the control group were collected between July 13, 2020 and September 5, 2020; those for the experimental group were collected between September 6, 2020 and October 31, 2020 after ad-ministering the resistance band exercise. To prevent exposure to treatment, evaluation in the experimental group followed that of the control group.

At baseline (week 0), data concerning general characteristics were collected. We measured the subjective and objective stress, lower extremity edema, and body composition at baseline and on the last day of the 4th and 8th week, after the end of the nurses’ shifts. The dependent variables were measured in both the experimental and control groups in the same place. Each participant was given an ID number, and data were coded accordingly, and the pretest and posttest data were analyzed.

### 2.6. Data analysis

The collected data were analyzed using IBM SPSS Statistics for Windows, version 26.0 (IBM Corp., Armonk, NY). The homogeneity of participants’ general characteristics was analyzed using frequency, percentage, mean, *X*^*2*^-test, and t-test, while the homogeneity of the dependent variables at baseline was tested using *t* tests. The effects of the experimental intervention were analyzed using a t-test and repeated measures analysis of variance. Further, the effect size between the independent and dependent variables was explained through partial *η*^²^ between group and time. A partial *η*^²^ of 0.01, 0.06, and ≥ 0.14 indicates a small, medium, and large effect size, respectively. Thus, the closer the partial *η*^²^ is to 1, the greater the difference in the means between groups and the smaller the respective error.^[35]^ The *P* value for statistical significance was set at *P* < .05.

### 2.7. Ethical consideration

Participants who elected to participate in the study were informed about the purpose and content of the study and a written consent form was signed by each participant, and the forms were kept by the researcher. The information sheet provided to the participants contained information about voluntary study participation and withdrawal, anticipated side effects, and treatment and compensation. The collected data were processed using ID numbers to protect personally identifiable information and were stored in a locked computer file after analysis. After data collection, the participants were offered vouchers for coffee stores as a token of appreciation. At the start of the study, TheraBand and elastic compression stockings were provided to the participants of the experimental group, and only elastic compression stockings were provided to the participants of the control group. All participants were provided with 20 to 30 mm Hg elastic compression stockings. The control group was provided with a TheraBand after the end of the experimental measurement.

### 2.8. Post-study treatment for the control group

After the completion of data collection in the experimental group, the same intervention was offered to the control group. Participants in the control group who preferred not to participate performed other exercises on their own. The same 8-week experimental intervention was provided to willing control group participants.

## 3. Results

### 3.1. Baseline homogeneity between the experimental and control groups

#### 3.1.1. Homogeneity of general characteristics and dependent variables.

There were no significant differences in age between the experimental (24.35 ± 1.64 years) and control group (25.33 ± 2.99), as well as in sex, length of career, current department, type of shift, exercise frequency, duration of standing work, work stress, smoking and drinking habits, use of medications, and current disease, confirming that the 2 groups were similar. There were also no significant differences in the dependent variables, namely subjective and objective stress, left and right calf circumferences, ankle circumferences, and muscle and fat thicknesses of the gastrocnemius and tibialis anterior, confirming the baseline homogeneity of the dependent variables between the 2 groups (Table 1).

**Table 1:** Homogeneity tests of subjects’ general characteristics (n = 44).

Characteristics	Category	Exp. (n = 23)		Cont. (n = 21)		*X*^²^ or *t*	*P*
		M or N	SD or %	M or N	SD or %		
Sex	Male	2	8.7%	4	19%	–.973	.337
	Female	21	91.3%	17	81%		
Age (year)		24.35	1.64	25.33	2.99	–1.338	.191
Career	< a yr	9	39.1%	9	42.9%	–.163	.872
	< 1 to 3 yrs	6	26.1%	3	14.3%		
	< 1 to 3 yrs	6	26.1%	7	33.3%		
	more than 5 yrs	2	8.7%	2	9.5%		
Department of work	MICU	9	39.1%	8	38.1%	–.173	.864
	SICU	5	21.7%	4	19.0%		
	TICU	9	39.1%	9	42.9%		
Frequency of exercise	< 3 times	17	73.9%	18	85.7%	.957	.344
	More than 3 times	6	26.1%	3	14.3%		
Standing time at work	< 6 h	0	0%	2	9.5%	2.295	.222
	More than 6 h	23	100%	19	90.5%		
Physical discomfort	Upper body	52	69.3%	43	59.7%		
	Lower body	23	30.7%	29	40.3%		
Stress degree	Little	13	56.5%	9	42.9%	.820	.547
	Many	10	43.5%	12	57.1%		
Stress (NRS)		6.30	1.46	6.14	2.54	.256	.800
Stress (Stress Index)		3.09	1.13	2.95	0.92	.432	.668
Calf Circumference (cm)	Lt	34.67	3.56	35.18	2.57	–.532	.598
	Rt	34.79	3.80	35.08	2.48	–.298	.769
Ankle Circumference (cm)	Lt	20.38	1.49	20.37	1.30	.016	.987
	Rt	20.61	1.70	20.36	1.27	.542	.591
Body composition (BPS) : Muscle thickness (mm)	Lt	62.42	12.34	61.85	13.71	.145	.885
	Rt	62.77	12.93	61.86	14.04	.222	.825
Body composition (BPS) : Fat thickness (mm)	Lt	10.13	2.02	10.25	2.10	–.190	.851
	Rt	10.45	1.85	10.67	2.12	–.366	.716

BPS = bodymetrix pro system, Con. G = control group, Exp. G = experimental group, Lt = left site, MICU = medical intensive care unit, NRS = numeric rating scale, Rt = right site, SD = standard deviation, SICU = surgical intensive care unit, TICU = trauma intensive care unit, cm = centimeter, mm = millimeter.

### 3.2. Effects of elastic band exercise on stress, lower extremity edema, and body composition

#### 3.2.1. Effect of elastic band exercise on subjective and objective stress.

Statistical analysis revealed that subjective stress was decreased in the experimental group than in the control group at 4 weeks (*t* = –1.770, *P* = .84; 95% confidence interval (CI): –0.957 to –0.063), significantly lesser at 8 weeks (*t* = –4.813, *P* < .01; 95% CI: –1.572 to –0.643).

NRS significantly differed according to time (*F* = 7.509, *P* = .004), group (*F* = 5.281, *P* = .011), and time and group interaction (*F* = 11.674, *P* < .001). Therefore, participants who used elastic bands had less subjective stress NRS than those in the control group. The partial *η*^²^, representing the effect of the elastic band exercise program according to time and group, was.217 (Table 2).

**Table 2:** Comparison of stress (NRS, stress index), left and right calf circumference, left and right ankle circumference, between the experimental and control groups.

Variable		Exp.(n = 23)		Cont.(n = 21)		t	*P*	*F*(*P*)
		Mean	SD	Mean	SD			
Stress (NRS)	Pretest	6.30	1.46	6.14	2.54	.256	.800	Time 8.791 (.002) Group 6.023 (.018) T*G 11.674 (<.001)
	Posttest 1	5.48	1.28	6.33	1.68	–1.910	.063	
	Posttest 2	3.96	1.43	6.33	1.68	–5.063	.000	
Stress (Stress Index)	Pretest	3.09	1.13	2.95	0.92	.432	.668	Time 2.137 (.136) Group 6.527 (.014) T*G 6.965 (.004)
	Posttest 1	2.70	0.70	3.14	0.96	–1.770	.084	
	Posttest 2	2.13	0.63	3.24	0.89	–4.813	.000	
Calf Circumference (Lt, cm)	Pretest	34.67	3.56	35.18	2.57	–.532	.598	Time 12.701 (<.001) Group .695 (.409) T*G 6.819 (.004)
	Posttest 1	34.29	3.61	35.01	2.50	–.769	.446	
	Posttest 2	33.91	3.75	35.06	2.53	–1.174	.247	
Calf Circumference (Rt, cm)	Pretest	34.79	3.80	35.08	2.49	–.296	.769	Time 14.827 (<.001) Group .154 (.697) T*G 8.280 (.001)
	Posttest 1	34.40	3.81	34.98	2.43	–.588	.560	
	Posttest 2	34.06	3.84	34.97	2.45	–.931	.357	
Ankle Circumference (Lt, cm)	Pretest	20.38	1.49	20.37	1.30	.016	.987	Time 24.340 (<.001) Group .154 (.697) T*G 6.194 (.006)
	Posttest 1	20.08	1.45	20.22	1.25	–.343	.733	
	Posttest 2	19.84	1.56	20.20	1.24	–.833	.410	
Ankle Circumference (Rt, cm)	Pretest	20.61	1.70	20.36	1.27	.542	.591	Time 36.448 (<.001) Group .021 (.886) T*G 15.287 < .001)
	Posttest 1	20.39	1.68	20.31	1.26	.183	.856	
	Posttest 2	20.12	1.62	20.26	1.27	–.307	.760	

Con. G = control group, Exp. G = experimental group, Lt = left site, SD = standard deviation, NRS = numeric rating scale, Rt = right site, T*G = time*group, cm = centimeter, mm = millimeter.

Statistical analysis revealed that objective stress was decreased in the experimental group than in the control group at 4 weeks (*t* = –1.910, *P* = .63; 95% confidence interval (CI): –0.855 to 0.480), significantly lesser at 8 weeks (*t* = −5.063, *P* < .01; 95% CI: –1.572 to –0.643).

Objective stress did not significantly differ according to time but differed significantly according to group (*F* = 6.527, *P* = .014) and time and group interaction (*F* = 6.965, *P* < .001). Therefore, participants who used elastic bands had less objective stress SI than those in the control group. The partial *η*^²^, representing the effect of the elastic band exercise program according to time and group, was 0.142 (Table 2).

#### 3.2.2. Effect of elastic band exercise on lower extremity edema.

▫ Effect of elastic band exercise on calf circumference

The left calf circumference significantly differed according to time (*F* = 12.701, *P* < .001) and time and group interaction (*F* = 6.819, *P* = .004), but not according to the group. However, there was no significant difference in the left calf circumference between participants who used the elastic band and participants of the control group. The partial *η*^²^, representing the effect of the elastic band exercise program according to time and group, was 0.140 (Table 2).

The right calf circumference significantly differed according to time (*F* = 14.827, *P* < .001) and time and group interaction (*F* = 8.280, *P* = .001), but not according to the group. However, there was no significant difference in the right calf circumference between participants who used the elastic band and participants of the control group. The partial *η*^²^, representing the effect of the elastic band exercise program according to time and group, was 0.162 (Table 2).

▫ Effect of elastic band exercise on the ankle circumference

The left ankle circumference significantly differed according to time (*F* = 24.340, *P* < .001) and time and group interaction (*F* = .6.194, *P* = .006), but not according to the group. However, there was no significant difference in the left ankle circumference between participants who used the elastic band and participants of the control group. The partial *η*^²^, representing the effect of the elastic band exercise program according to time and group, was 0.129 (Table 2).

The right ankle circumference significantly differed according to time (*F* = 36.448, *P* < .001) and time and group interaction (*F* = 15.287, *P* < .001), but not according to the group. However, there was no significant difference in the right calf circumference between participants who used the elastic band and participants of the control group. The partial *η*^²^, representing the effect of the elastic band exercise program according to time and group, was 0.267 (Table 2).

#### 3.2.3. Effect of elastic band exercise on body composition.

▫ Effect of elastic band exercise on muscle thickness

The left leg muscle thickness significantly differed according to time (*F* = 28.532, *P* < .001) and time and group interaction (*F* = 31.708, *P* < .001), but not according to the group. Therefore, participants who used elastic bands had a greater left leg muscle thickness than those in the control group. The partial *η*^²^, representing the effect of the elastic band exercise program according to time and group, was 0.430 (Table 3).

**Table 3:** Comparison of muscle and fat thickness between the experimental and control groups.

Variable		Exp. (n = 23)		Cont. (n = 21)		*t*	*P*	*F*(*P*)
		Mean	SD	Mean	SD			
Muscle Thickness (Lt, mm)	Pretest	62.77	12.93	61.86	14.04	.222	.825	Time 28.532 (<.001) Group 3.251 (.079) T*G 31.708 (<.001)
	Posttest 1	69.13	10.98	62.05	11.97	2.046	.047	
	Posttest 2	72.94	10.97	61.53	11.83	3.319	.002	
Muscle Thickness (Rt, mm)	Pretest	62.42	12.34	61.85	13.71	.145	.885	Time 22.421 (<.001) Group 1.983 (.166) T*G 18.630 (<.001)
	Posttest 1	67.64	10.27	62.59	11.96	1.507	.139	
	Posttest 2	71.02	9.97	62.18	11.40	2.746	.009	
Fat Thickness (Lt, mm)	Pretest	10.45	1.85	10.67	2.12	–.366	.716	Time 27.183 (<.001) Group 2.186 (.147) T*G 19.984 (<.001)
	Posttest 1	9.87	1.70	10.69	2.02	–1.469	.149	
	Posttest 2	9.17	1.59	10.58	1.89	–2.675	.011	
Fat Thickness (Rt, mm)	Pretest	10.14	2.02	10.25	2.10	–.190	.851	Time 13.438 (<.001) Group 2.264 (.140) T*G .24.640 (<.001)
	Posttest 1	9.43	1.62	10.44	2.12	–1.796	.080	
	Posttest 2	8.98	1.65	10.41	2.01	–2.603	.013	

Con. G = control group, Exp. G = experimental group, Lt = left site, SD = standard deviation, Rt = right site, T*G = time*group, cm = centimeter, mm = millimeter.

The right leg muscle thickness significantly differed according to time (*F* = 22.421, *P* < .001) and time and group interaction (*F* = 18.630, *P* < .001), but not according to the group. Therefore, participants who used elastic bands had a greater right leg muscle thickness than those in the control group. The partial *η*^²^, representing the effect of the elastic band exercise program according to time and group, was 0.307 (Table 3).

▫ Effect of elastic band exercise on fat thickness

The left leg fat thickness significantly differed according to time (*F* = 27.183, *P* < .001) and time and group interaction (*F* = 19.984, *P* < .001), but not according to the group. Therefore, participants who used elastic bands had a smaller left leg fat thickness than those in the control group. The partial *η*^²^, representing the effect of the elastic band exercise program according to time and group, was 0.322 (Table 3).

The right leg fat thickness significantly differed according to time (*F* = 13.438, *P* < .001) and time and group interaction (*F* = 24.640, *P* < .001), but not according to the group. Therefore, participants who used elastic bands had a smaller right leg fat thickness than those in the control group. The partial *η*^²^, representing the effect of the elastic band exercise program according to time and group, was 0.370 (Table 3).

## 4. Discussion

This study investigated the effects of an 8-week resistance exercise program on stress, lower extremity edema, and body composition in ICU nurses. We used a randomized control group pretest-posttest non-synchronized experimental design. After the 8-week elastic band exercise program, we observed that subjective and objective stress had decreased, muscle mass had increased, and fat mass had decreased. These findings suggest that elastic band exercise effectively improves workers’ quality of life in occupations that involve prolonged standing.

The fact that resistance exercises reduce stress and improve body composition balance, as observed in our study, implies that exercising has a positive psychological and physical influence, regardless of the method or duration of the exercise.

However, an unexpected finding was that there were no significant changes in the lower extremity edema. This could be because all the participants wore the same elastic compression stockings, regardless of the intervention. In this study, we measured the calf and ankle circumferences to examine the effects of the 8-week elastic band exercise program on lower extremity edema, but there were no significant changes. However, repeated measures analysis of variance showed that the calf and ankle circumferences significantly decreased over time, with significant time and group interaction effects. Therefore, subsequent studies should examine for changes in lower extremity edema after administering a resistance exercise intervention for a longer duration.

### 4.1. Strength and limitations of the study

The strengths of this study are as follows. First, we compared the composition of specific muscles and fat accurately using their thicknesses (mm) by including resistance exercises suitable for the lower extremity. Second, we analyzed sympathetic and parasympathetic ANS signals based on HRV to examine stress, a subjective emotion, using an objective parameter known as the stress index. Third, as this study was conducted during the COVID-19 pandemic, we created a video of the resistance exercise program and conducted non-face-to-face exercise education.

There are also some limitations to this study. First, we only included E University Hospital ICU nurses in D City; therefore, these findings cannot be generalized to all types of workers with prolonged standing. Second, the measurements were taken after each participant’s work shift; thus, exogenous variables related to time could not be controlled entirely. Finally, in this study, nurses in both the experimental and control groups wore elastic compression stockings during their shifts before participating in the intervention; therefore, there were limitations in examining the pure effects of resistance exercise on lower extremity edema.

## 5. Conclusions

This study is a randomized control group pretest-posttest non-synchronized experimental study investigating the effects of an 8-week elastic band exercise program on stress, lower extremity edema, and body composition in ICU nurses. The results showed that an 8-week elastic band exercise program influenced participants’ emotions and ANS, reducing subjective and objective stress, increasing muscle mass, and reducing fat mass. Thus, this confirms that resistance exercise using an elastic band positively affects individuals in the long term.

## Acknowledgments

We would like to thank all the participants and stakeholders for their assistance in conducting this study.

## Author contributions

**Conceptualization:** W.J.K., K.Y.K.

**Data curation:** W.J.K.

**Formal analysis:** K.Y.K.

**Funding acquisition:** W.J.K.

**Investigation:** K.Y.K.

**Methodology:** W.J.K.

**Project administration:** W.J.K.

**Resources:** W.J.K.

**Software:** W.J.K.

**Supervision:** K.Y.K.

**Validation:** W.J.K., K.Y.K.

**Visualization:** W.J.K.

**Writing – original draft:** K.Y.K.

**Writing – review & editing:** W.J.K.
